# Boosting motivation, cultural confidence, and native cultural awareness through digital storytelling in L2 language education

**DOI:** 10.3389/fpsyg.2026.1878796

**Published:** 2026-07-09

**Authors:** Xiaoxi Liang, Jinhui Liu, Liangshuai Wei, Weiwei Xu

**Affiliations:** 1 Huihua College of Hebei Normal University, Shijiazhuang, China; 2 School of Foreign Studies, Hebei Normal University, Shijiazhuang, China

**Keywords:** cultural confidence, digital storytelling, English-as-a-Foreign-Language curriculum, L2 Motivational Self System, native cultural awareness

## Abstract

With increasing emphasis on sustainability and cultural awareness in education, foreign language teaching is increasingly expected to extend beyond the introduction of target-language cultures alone. However, limited empirical research has explored how to incorporate students’ native cultural perspectives in a meaningful way into classroom teaching. Therefore, this study developed a Digital Storytelling for Native Cultural Awareness (DSTNCA) model based on social constructivism and the L2 Motivational Self System (L2MSS) to address this gap. The model will help Chinese English learners present their native culture with more confidence. A total of 281 undergraduate English majors participated in the study. The experimental group received DSTNCA intervention for 8 weeks, and the control group continued in regular classes. Results showed that DSTNCA increased students’ Ideal L2 Self motivation (β = 0.300, *p* < 0.01) and reduced Ought-to Self motivation (β = −0.247, *p* < 0.05). It also promoted a sense of national culture (β = 0.252, *p* < 0.01) and cultural awareness (β = 0.328, *p* < 0.01). Qualitative research indicated that digital storytelling enabled students to view themselves as “cultural ambassadors” and strengthened their sense of identity and self-confidence through English study. The above results suggested that integrating local culture and history in English-as-a-Foreign-Language curriculum development can make learning more meaningful and inspire students to continue their studies voluntarily.

## 1 Introduction

**L**anguage learning in foreign language education refers to the acquisition of language knowledge and skills, as well as the development of intercultural communicative competence (Deardorff, 2017; Tang et al., 2024). For Chinese learners of English, this means understanding English-speaking cultures while also gaining the ability to present and explain Chinese culture in English. However, many English curricula still focus more on content from Anglophone countries, and Chinese cultural content is often given less attention and is sometimes omitted. Some scholars have referred to this imbalance as a “noticeable omission” of native culture in foreign language education (Banaruee et al., 2023; Liu et al., 2023; Lu et al., 2022).

This issue is crucial for learners’ intercultural studies. When students have a lack of Native Cultural Awareness (NCA), they may be unable to present Chinese cultural ideas, traditions, values, and daily cultural practices in English (Wu and Miller, 2021; Yang, 2018). Therefore, cross-cultural communication will be one-way, as students may know how to talk about Western festivals and social norms, but feel less confident in explaining their own culture in response to similar questions (Akizhanova, 2021). This lack of confidence may also reduce their motivation to learn English and make the learning process less meaningful in relation to their own culture (Boo et al., 2015; Foreign Language Teaching and Research Press, 2000; Wang and Liu, 2010).

Therefore, the study introduces and evaluates a Digital Storytelling of Native Cultural Awareness (DSTNCA) model. Drawing on social constructivism, the model is a collaborative digital storytelling-based active learning method. Students will explore and present short digital stories about Chinese culture in English through cooperative research and writing. These stories are usually in the form of two- to five-minute videos that combine narration, pictures, sound, and other multimedia. Thus, students are able to learn Chinese by connecting it with their own cultural and develop greater confidence to present Chinese culture.

Dörnyei’s (Dörnyei, 2009) L2 Motivational Self System (L2MSS) were also employed in this study to explore the role of DSTNCA in motivating students. Digital storytelling is a collaborative and creative way to help students feel that they are more capable intercultural communicators, enhance their Ideal L2 self-concept, and increase their awareness of native culture. Therefore, this study investigates whether the DSTNCA model can enhance the NCA ability of Chinese foreign language learners and examines whether such an improvement is accompanied by changes in their foreign language learning motivation. Based on the previous studies, the study intends to provide a practical model for promoting students’ sense of cultural confidence in foreign-language classes.

## 2 Literature review

### 2.1 Digital storytelling in pedagogical application

Digital storytelling (DST) has been increasingly adopted in FL classrooms. It often requires students to create short personal stories by combining images, written text, recorded voice, music, and other digital elements into a coherent narrative (Lambert, 2013). DST offers more opportunities for active participation and agency in learning for students than traditional classroom activities. Rather than being passive receivers of information or completing language exercises, students are required to plan, select, organize, and present ideas in language and multimedia. Thus, DST may be more interesting and practical for language learning, helping to motivate students more strongly, think critically, and study in a real-life context.

DST can be employed in FL learning to connect language use with personal expression. When DST is added to the classroom activities, students will have more opportunities to participate in and use the target language authentically (Hava, 2021). The creation process of a digital story can also boost students’ self-confidence in speaking and writing, as they will be using language to express their own ideas that are meaningful to them rather than just learning grammatical patterns. DST uses visuals, words, and other kinds of personal factors to interest learners in several ways and thus helps them learn language more deeply and remember it longer (Kim, 2014; Lotherington and Jenson, 2011).

Previous research has shown that DST and the drive to learn are related. According to Kasami (Kasami, 2021) and Coggin (Coggin et al., 2019), DST tasks can help to boost students’ self-confidence and motivation, and these are closely related to Dörnyei’s (Dörnyei and Ushioda, 2009) L2 Motivational Self System (L2MSS), particularly the development of the Ideal L2 Self. During the process of DST training, students are guided to reflect on their language skills, personal experiences, and sense of identity as language users. The above reflection may motivate them to learn the target language better and longer. In addition, DST also turn the students into active content creators. In their design and production of stories, they need to collect information, make choices, organize materials, and evaluate the effectively of the message transmission. These activities can help cultivate higher-order thinking skills of analysis, synthesis, and evaluation (Barrett, 2006; Braun and Clarke, 2006). Students can also participate in the DST project more actively and learn to reflect on their own learning from it more deeply (Harjono and Wiryotinoyo, 2021; Sadik, 2008).

However, many of the existing studies on DST have focused mainly on language development, learner engagement or technological skills. Less attention has been paid to how DST may support cultural understanding and cultural self-confidence for learners who need to present their own native culture in an international context. This issue is particularly prominent for Chinese FL learners, as their English curricula often place more emphasis on Anglophone cultures and provide fewer chances for students to explore and present Chinese cultural heritage in English (Banaruee et al., 2023). In light of the above deficiencies, this study introduces the Digital Storytelling for Native Cultural Awareness (DSTNCA) model to enhance the level of Native Cultural Awareness (NCA) in Chinese FL learners through DST.

### 2.2 Native cultural awareness in culturally responsive foreign language teaching

Culturally responsive teaching in FL pedagogy has received increasing attention recently as teachers begin to consider learners’ own cultural backgrounds in their lessons, not just the culture of the target language. In many traditional FL classrooms, cultural teaching has mainly been conducted by original textbooks, films, television materials, and role-play dialogues in the target-language context. Although these materials are convenient, they may also lead to an unbalanced distribution of culture. Learners are often exposed to English-speaking cultures in depth, but their own native culture receives relatively less attention (Tang, 2004). This may result in the same problem for Chinese learners: although they can discuss Western festivals, customs, or social situations in English, they are still unable to express Chinese cultural experiences, values, or stories in the same language. Hu (Hu and Bentler, 2022) has also pointed that learners are unable to express their own culture in the target language. Therefore, foreign language education should create space for both target culture and native culture, rather than treating the latter as an additional or secondary subject.

NCA is also suitable for this. Based on Byram’s (Byram, 1997) model of ICC, NCA can be divided into four related dimensions: attitudes, knowledge, skills, and critical cultural awareness. Attitudes refer to openness and curiosity toward both one’s own culture and other cultures. Knowledge involves understanding social groups, cultural products, and daily practices. Skills are the capacity to understand, compare, discover, and communicate in a multi-cultural contexts. Critical cultural awareness refers to the capacity to critically evaluate different cultural ideas rather than passively accepting them (Baker, 2012).

Previous studies have suggested that intercultural awareness is heart of ICC (Kramsch, 1993; Wang et al., 2021). Kramsch (Kramsch, 1993), for example, questioned the traditional idea that learners should simply “do as the Romans do” and proposed instead that intercultural learning should involve dialogue, reflection, and negotiation between the two cultures. This view is especially suitable for Chinese FL education, where students need to learn English-speaking cultures and present Chinese culture clearly and confidently in English (Fan, 2022; Wu, 2021). Fan’s (Fan, 2022) production-oriented approach provides supporting evidence for this study and suggests that adding Chinese stories to English classes can boost students’ confidence in their national culture and promote all-round intercultural communication.

However, some questions have not yet been solved in recent years. Many of the existing studies on intercultural communication are relatively general in scope, and fewer studies have explored how classroom teaching can motivate learners actively express their native culture through the foreign language. Many studies continue to focus on improving students’ understanding of the target culture, but pay less attention to share their own cultural background confidently. While research has been carried out on cultural confidence and intercultural awareness, there is still a need for empirical studies of practical teaching models in the Chinese FL context.

Therefore, the study introduces DSTNCA model. Students create digital stories of Chinese culture in English according to this model. This process gives them a practical opportunity to research, organize, and present native cultural content through the target language. It also helps them build a stronger sense of Chinese culture and develop intercultural communicative competence more actively and personally.

### 2.3 Social constructivism as a theoretical foundation

The DSTNCA model is based on social constructivist theory, which helps explain why collaborative digital storytelling can support foreign language learning. As both an educational and psychological theory, Social constructivism emphasizes that learning occurs in society through social interaction and cultural context (Derry, 1999; McMahon, 1997). Therefore, knowledge is not simply transferred from teacher to student or acquired by learners independently, but is constructed through communication, cooperation, and shared activities within a social context (Wibowo et al., 2025). Vygotsky (1978) argued that learning first takes place through interaction with others and is later internalized by the individual, distinguishing between the interpsychological and intrapsychological levels. This view offers the foundation for applying collaborative storytelling activities in the foreign language classroom.

Several ideas from social constructivism have been incorporated into the design of DST activities. One of the most important is collaborative learning, which aims to help students build knowledge together. Previous studies have shown that collaborative learning can support students’ cognitive development and problem-solving abilities (Amineh and Asl, 2015; Van Meter and Stevens, 2000). Students do not study language independently in DST projects. They discuss cultural topics, decide how to structure their stories, revise scripts, choose suitable images or sounds, and negotiate how meaning should be presented. The above interactions provide students opportunities to use English in practical communication and learn about the culture . Thus, learners can develop language skills, critical thinking, and creativity while engaging the linguistic and cultural demands (Nair and Yunus, 2021).

Another useful idea from Vygotsky’s theory is the Zone of Proximal Development (ZPD), which is the range of learning that can be achieved by a child with the help of more knowledgeable others, but not yet by themselves. Scaffolding is based on the above idea and provides students with temporary support to help them learn how to do it independently (Wood et al., 1976). Such support in digital storytelling may take various forms. Teachers provide models, examples, and feedback, while peers support and structured tools such as story templates, script outlines, and multimedia guidelines help students complete the task more effectively. Emotional support plays an important role, as students may need encouragement when presenting their own culture in English. These kinds of scaffolding help students create English narratives on Chinese culture and develope their linguistic ability and cultural awareness (Fu, 2018; Meyer and Turner, 2007).

The DSTNCA model in this paper will use the ideas of social constructivism to build a classroom environment that promotes student learning through cooperation, guided practice and digital creation. In addition, peer collaboration, teacher guidance, and multimedia tools together help students construct knowledge, express native cultural meanings, and improve their English communication skills. Rather than presenting culture as background, the model motivates students to actively learn about Chinese culture and express it through the target language.

## 3 Research methodology

### 3.1 Research questions

Based on the above discussion, this study focuses on two research questions:

(1) To what extent does the DSTNCA model influence FL learners’ L2 Motivational Self System compared with conventional instruction?
(2) To what extent does the DSTNCA model influence FL learners’ Native Cultural Awareness compared with conventional instruction?

### 3.2 Instructional setting

This study has introduced the design and classroom application of DSTNCA model. The model was added to an “Integrated English” course for 281 second-year English majors in China. Its main purpose was to promote the development of students’ intercultural communicative abilities in language teaching. Instead of having students only to read, listen, or respond to prepared materials, the DSTNCA activities invited them to create their own cultural stories in English.

The model had the following two differences from typical digital storytelling tasks. First, the content of the activities considers what students’ daily life is on the Internet. Many Chinese university students are familiar with short-video platforms such as Bilibili and Douyin, where stories are often presented through brief, visually engaging videos (Gao, 2021). Following this structure, students were asked to produce short videos of about two to 5 min for their classmates. During video production, students employed accessible editing software and integrated multimodal resources, such as narration, images, subtitles, and background music, to create more vivid representations of their stories.

Second, the DSTNCA model provided students with a relatively free-form production system. Within the general cultural themes, they could select particular stories they want to share, choose suitable digital tools, and develop their own modes of presentation. Therefore, this form is more convenient and can be better integrated with students’ interest in culture to make the work more meaningful. Through this process, students were able to see themselves as English learners who could present Chinese culture in their own ways.

### 3.3 Participants

The quantitative part of the study included eight classes of second-year undergraduate English major students from a comprehensive university in Hebei Province, China. All participants were in the same year level and followed a standardized English major curriculum required by national higher education requirements. Before attending university, they had received about 10 years of English classes.

A total of 281 students participated in the study, including 111 males (39.50%) and 170 females (60.50%). Most students were 19 or 20 years old, with 58 students aged 19 (20.64%) and 122 aged 20 (43.42%). Smaller numbers of students were aged 18 (*n* = 2, 0.71%), 21 (*n* = 31, 11.03%), and 22 (*n* = 5, 1.78%). Collect the background information to describe the sample and check whether the groups can be generally compared before the intervention.

Randomly select four classes from the entire group for the intervention study. Two classes in the experimental group had *n* = 140, and the two classes that were not included were the control groups (*n* = 141). Therefore, the final sample size was 281 students. This number was deemed suitable for the study design, as it was significantly higher than the general minimum of 25 participants per group required for research on educational intervention (Horgas and Miller, 2008; Kelley, 2007). An expanded sample size will reduce the probability of a Type II error and improve the power to distinguish between the two groups. Nine students were invited to take part in follow-up interviews after the quantitative phase, as shown in Table 1.

**TABLE 1:** Background characteristics of interviewees.

Serial Number	Interview code	Gender	Age
1	GYR	Female	20
2	LQH	Male	19
3	LXY	Female	20
4	LDL	Male	21
5	XSJ	Female	20
6	YM	Female	20
7	ZSY	Female	19
8	ZS	Female	21
9	ZL	Female	20

### 3.4 Research design

This study adopted a sequential explanatory mixed-methods design (Creswell and Plano Clark, 2023), with a quasi-experimental component embedded in the first phase. The design was employed to measure the effects of the DST-based intervention from the students’ perspectives.

The quantitative phase involved comparing the experimental and control groups. The experimental participated in the DSTNCA activities, and the control group studied the same textbook units for conventional teacher-led instruction, including text analysis, classroom presentations, and language exercises. No digital storytelling activities were included for the control group. After the quantitative results were obtained, qualitative data were collected to provide a fuller interpretation of the findings. Thus, the design could be used to observe how much the DSTNCA model achieved in terms of measurable results and to explore the learning experiences behind those outcomes.

### 3.5 Research instruments

Quantitative and qualitative data were collected in this study to examine the impact of DSTNCA intervention on students’ motivation and sense of native culture. For the quantitative part, students completed Chinese versions of the L2MSS questionnaire (Boo et al., 2015; Liu, 2012) and the Native Cultural Awareness questionnaire (Wu and Miller, 2021) in both the pre- and post-intervention periods. These two questionnaires were used to observe changes in students’ language learning motivation and their consciousness of native culture.

The three parts of the online pre-test are shown below. The first part collected basic demographic information from students in both the experimental and control groups. The second part measured students’ L2 Motivational Self System through 14 items on a six-point Likert scale, ranging from 1, “completely disagree,” to 6, “completely agree.” One sample item was: “If I participate in the activities of telling Chinese stories in English, I will come up with creative ideas.” The third part focused on native cultural awareness. Higher scores indicated stronger or more positive native cultural awareness.

Nine students from the experimental group were selected purposefully for follow-up interviews and qualitative data were collected. Semi-structured interviews and reflective journals were employed to explore students’ experiences during the intervention in more depth. The data show that students’ motivation, confidence, and cultural identity have all changed differently in relation to the English digital story activity. The results from questionnaire and qualitative data provided all-around evidence of the actual function of the DSTNCA model.

### 3.6 Intervention procedure

The DSTNCA intervention lasted 8 weeks and had three cycles. Each round was linked to a thematic unit from Contemporary College English, written by Yang Limin and published by Foreign Language Teaching and Research Press. The textbook provided the basic classroom content and the DSTNCA tasks extended this by having students learn about related Chinese cultural themes in English. Thus, the intervention was connected to the regular curriculum rather than added as a completely separate activity.

**FIGURE 1:**
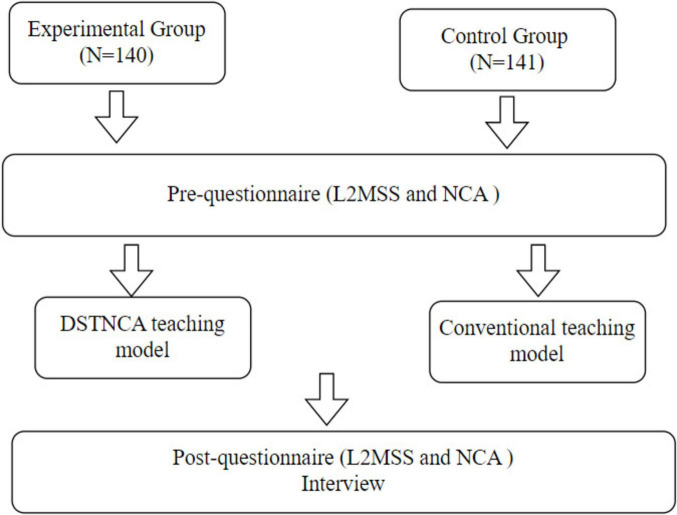
The three-stage DSTNCA operational model.

The three rounds focused on the following topics: Chinese Wisdom based on Unit 2, Chinese Landscapes and Cultures based on Unit 4, and Chinese Moral Stories based on Unit 9 (Xie, 2022). As shown in Figure 1 and explained in Table 2, each round followed a three-step process. The process guided students from initial cultural exploration and story planning to digital story production and final reflection.

**TABLE 2:** Week-by-week teaching D\design of the DSTNCA model.

Week	Theme (unit)	Activities (stage)	Participants	Instruments/ output	Data collection
1	All	Pre-test Questionnaires (L2MSS and NCA)	Students	Questionnaires	Quantitative (Baseline)
2	Chinese Wisdom (Unit 2)	Stage 1: Preparation and Video CreationStage 2: In-class Sharing and DiscussionStage 3: Reflective Journal Writing	Students	Short videos;Peer feedback;Reflective journals;	Qualitative
3 and 5	Chinese Landscapes (Unit 4)	Stage 1: Preparation a Video CreationStage 2: In-class Sharing and DiscussionStage 3: Reflective Journal Writing	Students	Short videos;Peer feedback;Reflective journals;	Qualitative
4 and 6	Chinese Tradition (Unit 4)	Stage 1: Preparation and Video CreationStage 2: In-class Sharing and DiscussionStage 3: Reflective Journal Writing	Students	Short videos;Peer feedback;Reflective journals;	Qualitative
7	Chinese Moral (Unit 9)	Stage 1: Preparation and Video CreationStage 2: In-class Sharing and DiscussionStage 3: Reflective Journal Writing	Students	Short videos;Peer feedback;Reflective journals;	Qualitative
8	All	Post-test QuestionnairesSemi-structured Interviews	All StudentsPurposive Sub-sample	Questionnaires;Interview; Transcripts;	Quantitative and Qualitative

By contrast, the students in the control group also studied the same three textbook units, Units 2, 4 and 9, under traditional classroom teaching. The learning activities of the students included teacher-led text explanation, textbook exercises, presentation preparation, and regular journal writing. No digital storytelling tasks were included in the control group.

Overall, the intervention was designed to have students experience the native-speaking culture repeatedly, encourage them to use English in expression naturally, and make them reflect on what they have learned after each round of digital story creation.

Stage 1: Preparation and Digital Video Creation (Pre-class)

Before class, students worked individually or in groups to make a two- to five-minute video of a Chinese story in English. They first selected a topic from the given theme and searched for relevant background information. Then, they wrote an English script and paid attention to both linguistic accuracy and the cultural significance of the story. Students were familiar with CapCut and Jianying smartphone editing applications in the production stage. Combine narration and pictures to tell the stories through various ways, such as short videos or background music.

Stage 2: In-class Sharing and Discussion (In-class)

Students shared their videos with their classmates in class. The classroom is now an interactive space for presentation, discussion, and group learning. After watching a video, the students, under the teacher’s guidance, would have a discussion on what they learned about the content and language use in the video. The students had a genuine audience for their English storytelling and could also receive feedback from their classmates and the teacher at this stage. It also provided a platform for students to compare various forms of representation for Chinese culture in English.

Stage 3: Reflective Journal Writing (Post-class)

After each round, students wrote a short reflective journal in English of about 200 words. The journal followed a structured of Farrell’s reflective framework and adapted it (Farrell, 2013). Students were asked to describe the main content of their own video and their classmates’ videos, explain how the storytelling task related to the textbook unit and their previous knowledge, and reflect on any changes in their motivation to learn English and their awareness of native culture. They were also motivated to consider how to make these changes in the next round.

This three-stage cycle was repeated multiple times in the 8 weeks. Through repeated preparation, sharing, discussion, and reflection, students had continuous chances to use English in the exploration and presentation of Chinese culture.

### 3.7 Data analysis

The quantitative data were analyzed with STATA. Paired-sample *t*-tests were first used to compare students’ scores before and after the intervention. Multiple regression analysis was then conducted to examine whether the DSTNCA model was associated with changes in students’ motivation and native cultural awareness. These analyses helped identify the extent to which students’ L2MSS and NCA changed over the course of the intervention.

The first is the contents of semi-structured interviews and reflective diaries. These materials were analyzed through thematic analysis and support by ATLAS.ti 25 (Braun and Clarke, 2006). Coding employed both deduction and induction in the process. Some codes were developed from the existing concepts of L2MSS and NCA, and others were added due to new patterns in the data. Therefore, it is now more feasible to study students’ experiences in more detail, including how they have felt about their motivation, sense of cultural confidence, and learning of native culture during the DSTNCA activities.

## 4 Results

### 4.1 Preliminary analysis

The reliability test showed that the L2MSS questionnaire had good internal consistency. The entire Cronbach’s alpha was 0.838. Among the three dimensions, Ideal L2 Self had an alpha value of 0.913, and L2 Learning Experience reached 0.935, indicating very strong consistency. The Ought-to L2 Self dimension also showed acceptable reliability, with a Cronbach’s alpha of 0.830. The suitability of the data for factor analysis was examined through the Kaiser-Meyer-Olkin (KMO) test. The overall KMO value for the L2MSS questionnaire was 0.805, suggesting that the data were appropriate for factor analysis. Among the dimensions, L2 Learning Experience had the highest KMO value, at 0.868.

The NCA questionnaire also showed satisfactory reliability. The Cronbach’s alpha values were 0.804 for Attitude, 0.820 for Awareness, and 0.772 for Skills and Knowledge. The overall Cronbach’s alpha for the pre-test questionnaire was 0.867, showing good internal consistency. The KMO value was 0.733, and thus the data met the requirements for further factor analysis. These results suggest that both questionnaires were reliable and suitable for use in this study.

### 4.2 Quantitative results

To examine the effect of the DSTNCA intervention, statistical analyses were conducted to compare the experimental group (EG), which participated in DSTNCA activities, with the control group (CG), which received regular instruction. A change in each group was made from the pre-test to the post-test, and the difference between the two groups after intervention was also noted.

#### 4.2.1 Pre-post intervention changes

The descriptive results showed that both groups improved from pre-test to post-test, although the forms of improvement differed between them, as shown in Table 3. Therefore, the two types of instruction may have affected students differently.

**TABLE 3:** Descriptive statistic and effect sizes for pre-post changes.

Variable	Group	Pre-test mean	Post-test mean	Mean difference	Cohen’s d	Magnitude
Motivation - Ideal Self	EG	2.62	3.22	**+0.60**	**−0.59**	Medium
	CG	2.82	3.12	+0.30	−0.35	Small
Motivation - Learning Experience	EG	2.91	3.36	**+0.45**	**−0.40**	Small/Medium
	CG	3.06	3.40	+0.34	**−**0.33	Small
NCA - Attitude	EG	2.65	3.11	**+0.46**	**−0.63**	Medium
	CG	2.82	3.03	+0.21	**−**0.38	Small
NCA - Awareness	EG	2.72	3.21	**+0.49**	**−0.59**	Medium
	CG	3.01	3.17	+0.16	**−**0.25	Small
NCA - Skills	EG	2.52	3.02	+0.50	**−**0.48	Small/Medium
	CG	2.72	3.39	**+0.67**	**−0.60**	Medium
NCA - Knowledge	EG	2.42	3.12	**+0.70**	**−0.98**	Large
	CG	2.56	3.14	+0.58	**−**0.71	Medium/Large

Bolded entries highlight Cohen’s *d* values and mean differences that reached at least a medium magnitude (≥0.50), indicating practically meaningful pre to post improvements. All effect sizes are positive; the underscores (‘_’) preceding *d* values are typographical artifacts and do not denote negative signs.

Paired-sample *t*-tests showed that most of the changes in the EG were statistically significant at the *p* < 0.001 level. The only exception was Ought-to Self motivation, which did not change significantly (*p* = 0.922). This finding suggests that the DSTNCA activities are likely more motivated by students’ own interests and a sense of cultural identity rather than by external pressures or demands. EG showed good progress in Ideal Self motivation, Cultural Attitude, and Cultural Awareness, which were in line with the aims of the intervention.

EG was also relatively higher than CG in the effect size of this section. Therefore, it can be seen that the DSTNCA model may have benefited students more in forming an interest in English study and developing a sense of culture. The increase in Skills was lower for CG. This may be due to the regular curriculum, which has placed more emphasis on textbook-based practice of targeted language-skill development. However, the DSTNCA model also seems to have promoted all kinds of development by linking language use with cultural awareness and individual expression.

#### 4.2.2 Between-group differences post-intervention

The EG performed better than the CG in many places after the intervention, as shown in Table 4. First, EG was significantly better than the control group in Ideal Self-motivation; the effect size was medium (Cohen’s d = 0.45) and *p* = 0.034. This result suggests that participation in DSTNCA activities may have given students a stronger sense of self-confidence and self-esteem as English learners.

**TABLE 4:** Post-intervention group differences.

Variable	Mean Difference (EG - CG)	*p*-value	Cohen’s d	Interpretation
Motivation - Ideal self	+0.10	0.034	**0.45**	Medium (EG Higher)
NCA - Attitude	+0.08	<0.001	**0.39**	Small/Medium (EG Higher)
NCA - Awareness	+0.04	<0.001	**0.42**	Small/Medium (EG Higher)
NCA - Skills	−0.37	<0.001	−0.31	Small (CG Higher)
NCA - Knowledge	−0.02	0.890	−0.02	Negligible

Bolded entries denote statistically significant mean differences between the experimental and control groups (*p* < 0.05). Cohen’s *d* values preceded by an underscore (‘_’) are negative, indicating that the post test scores of the control group were higher. Magnitude descriptors follow Cohen’s (1988) conventions (small ≥ 0.20, medium ≥ 0.50, large ≥ 0.80).

EG also showed an increase in Cultural Attitude, but it had a relatively small to medium effect size, Cohen’s d = 0.39, and *p* < 0.001. Therefore, the students are more inclined to learn culture now after participating in the experiment. The EG was also better than the CG in Cultural Awareness, but the effect size was small to medium (Cohen’s d = 0.42, *p* < 0.001). Therefore, the DSTNCA activities have helped students better understand the culture, difference and presentation of Chinese in English.

These findings indicate that the DSTNCA model had a stronger effect on students’ Ideal Self motivation, Cultural Attitude, and Cultural Awareness than regular instruction. Furthermore, digital storytelling may help students connect English learning with their cultural identity and extend learning environment outside the classroom.

### 4.3 Qualitative results

The qualitative data helped explain how students participated in the DSTNCA activities. Collectively, the interviews and reflective journals showed that the model helped students learn more actively, work together better, and gained confidence in speaking English about Chinese culture.

Many students described the story-making process was in a group. Together they selected the topic, wrote the script, practiced pronunciation, filmed and edited. Peer support helped reduce the students’ stress in learning and boosted their self-confidence in using English. One participant explained, “Working with peers gave me confidence—when I made mistakes in pronunciation, my group members helped me correct them patiently. We laughed and fixed the sentences together” (Participant LDL, Interview). A student further added that the project promoted independent exploration: “I am able to independently learn various traditional fairy tales by searching for information online and using library resources. This project made me curious about the historical background” (Participant LXY, Interview).

The team project has also increased the sense of achievement and self-efficacy among the students. Students had to complete a language task, but they also needed to solve real-life problems in their group, such as how to present the story clearly and divide group responsibilities to enhance the group’s interest in making a video. One of the participants said that “After successfully working with my group to film and edit our scenes, I came to appreciate the value of teamwork as a cultural trait more deeply” (Participant LXY, Reflective Journal). Some students also received positive feedback from their peers and thus felt more capable. For example, one student said, “The positive feedback I received from my group after presenting made me feel competent and suddenly more interested in the culture we were studying” (Participant LDL, Interview). Another participant was able to feel that they were getting better at English through this project and were therefore less anxious about learning challenging cultural concepts, as shown in their reflective journal (Participant ZL).

The Digital Storytelling activity has helped students learn more about their own culture at home. Through research, selection and presentation of Chinese cultural stories, the students started to see them as cultural resources they could share with others. One participant said that after watching the finished video showing a traditional Chinese value, he or she felt very proud of it (Participant ZL, Interview). Another student wrote that he felt a sense of pride and was able to present their work more confidently because he knew it came from his own background (Participant LXY, Reflective Journal).

Some students also linked their cultural confidence with the technical skills they developed during the project. Learning to edit videos and add subtitles has made it easier to present Chinese culture in a modern, accessible way for more people. As one participant explained, “I was more likely to feel a sense of achievement by learning a new way of editing to present the cultural stories more visually. With the above skills, I believe I will be able to present Chinese culture in a new form” (Participant LDL, Interview).

The qualitative findings indicate that the DSTNCA model provided more support for language practice. Through collaborative work, digital production and cultural storytelling, students have been more actively involved in learning and are now more confident using English to convey Chinese cultural ideas. The findings also indicate that by participating in the storytelling of our hometowns, students will be able to feel a sense of belonging and be more confident using English.

## 5 Discussion

The DSTNCA model is expected to boost the enthusiasm for learning and cultural awareness among Chinese FL learners. Based on both the quantity and quality of the results, the two groups showed the same trend: the DSTNCA group had higher ideal L2 self-motivation and made significant gains in the affective aspects of NCA, specifically attitude and awareness. Four characteristics of the model that can be inferred from the above modifications are student agency, collaboration, authentic task design and the application of familiar digital tools.

A typical feature of the DSTNCA model is that it changed the role of students in cultural learning. Instead of passively receiving cultural knowledge from textbooks and teachers, students had to select, comprehend and demonstrate this culture independently. This reflects the basic idea of active learning, in which students are guided to learn by themselves through participation rather than simply receiving knowledge passively (Bonwell and Eison, 1991; Reilly and Reeves, 2024). The increase in Ideal L2 Self motivation may be related to this change in role (Thomas et al., 2024). Students’ learning was no longer about acquiring cultural knowledge; they began to believe that they were capable of sharing Chinese culture in English. The interview data also show the same. One participant said, “I dream of being a cultural ambassador one day. This project was the first experience I had. I became interested in learning more about the rich and wonderful culture of our country to spread it” (Participant LXY, Interview). This comment shows that through the creation process of this work, a new learning method has been established.

Collaboration was another important part of the DSTNCA model. Based on Vygotsky’s social constructivist view, the activities had students collaborate in the planning, writing, filming, and modifying their digital stories (Vygotsky, 1978). In this process, peer support helped students deal with language difficulties and reduced some of the stress they experienced in using English (Hakim and Wingate, 2025; Lock and Redmond, 2021). Peer can help each other learn and complete study groups that are more difficult to arrange alone. This was especially clear in the theme of “Developing Confidence Through Peer-Supported Storytelling Tasks.” For example, one student explained, “Working with classmates made me confidence. When I made mistakes in pronunciation, my group members kindly corrected me” (Participant LDL, Interview). This kind of peer support is in line with the concept of the Zone of Proximal Development, where learners are able to reach a higher level of performance with help from others (Liu et al., 2024).

The design of the learning activity also seemed to matter. The DSTNCA activities were based on the idea of “telling China’s story well,” and thus the students had a stronger motivation to use English. After completing the exercises in the textbook, the students still needed to prepare cultural stories for a live performance in their own right, even if it was only for a small-group class. It has expanded the learning opportunities for students and motivated them to consider in depth how Chinese culture can be presented to others. The above authenticity may be the reason for the increase in Cultural Attitude and Cultural Awareness (Lin and Wang, 2021). As one participant noted, “I was able to spread the culture abroad more widely through the use of digital media more effectively than before” (Participant LDL, Interview). This suggests that students began to perceive the use of English in the context of culture as having other purposes than academics.

Technology also had a certain practical purpose for the model. The students are familiar with the tools and thus use existing video-editing software and short-video platforms. As these tools were used every day online, they did not feel like an extra burden. Instead, they offered students many more structures and expressions for their cultural stories. This may have reduced the pressure students usually feel about producing in a foreign language (Krashen, 1989), and increased their willingness to participate in the task (Cui, 2025). Some students also felt that they had acquired new technical skills and were more capable. One participant said, “I had achieved the difficult task of learning a new editing method to present our culture more attractively. This technical ability made me feel more capable of representing my culture in a modern way” (Participant LDL, Interview). Therefore, technology supported both language use and cultural expression.

## 6 Conclusion

DSTNCA model was developed and studied in this paper to connect foreign language teaching with native culture. The quantitative results showed that the model positively influenced students’ Ideal L2 Self motivation and helped reduce their Ought-to Self motivation. And also promoted changes in the affective elements of native cultural awareness, such as attitudes and Awareness. These results were supported by the qualitative findings, which showed that students gradually began to believe that they could be “cultural ambassadors.” Through researching, creation and presentation of Chinese cultural stories in English, they became more confident in expressing their own culture and more aware of the value of cultural communication.

The findings also suggest that promotional motivation played an important mediating role in this process. It helped explain how the DSTNCA activities affected all parts of native cultural awareness among Chinese FL learners. In other words, students’ higher desire to be positively portrayed in English was believed to have increased their cultural confidence and awareness.

The study offers several implications for foreign language education. Theoretically, the DSTNCA model supports Dörnyei’s (Dörnyei, 2019) L2 Motivational Self System by showing that students’ motivation to learn a foreign language can be strengthened when language learning is connected with cultural identity. Instead of focusing on motivation only as a desire to master the target language, this study suggests that learners may also be motivated by the wish to represent their own culture through that language. The model also reflects Vygotsky’s (Vygotsky, 1978) social constructivist theory of learning, and students construct their knowledge in collaboration with peers through support and discussion on storytelling tasks.

In practical terms, the DSTNCA model provides a useful approach for teachers who want to combine language learning with cultural awareness. By asking students to create digital stories about native culture, the model gives learners a more active role in the class. Students’ participation extends beyond the completion of language exercises, as they actively select cultural topics, develop narratives, use digital tools, and present their work to others. This will help the students practice the language, experience culture, interact with others, and create. Reflective journals and peer feedback can help students better reflect on their learning in English and culture.

However, this study has several limitations. First, the participants were English majors in the undergraduate department of one university, and the sample was not fully random. Therefore, the results may not be applicable to all groups of learners. Future studies could use randomized controlled designs and include students from different educational levels, regions, and cultural backgrounds. Second, the intervention lasted only 8 weeks, so the long-term effects of the DSTNCA model has not been observed. A longitudinal study can be carried out to determine whether the students’ motivation and awareness of native culture have been sustained after the intervention period. Finally, this paper studied Chinese FL learners. Further studies can apply this model in other cultures and language-learning scenarios to determine if it is suitable for learners outside of China.

## Data Availability

The original contributions presented in this study are included in this article/supplementary material, further inquiries can be directed to the corresponding author.
